# *PUF60* variants cause a syndrome of ID, short stature, microcephaly, coloboma, craniofacial, cardiac, renal and spinal features

**DOI:** 10.1038/ejhg.2017.27

**Published:** 2017-03-22

**Authors:** Karen J Low, Morad Ansari, Rami Abou Jamra, Angus Clarke, Salima El Chehadeh, David R FitzPatrick, Mark Greenslade, Alex Henderson, Jane Hurst, Kory Keller, Paul Kuentz, Trine Prescott, Franziska Roessler, Kaja K Selmer, Michael C Schneider, Fiona Stewart, Katrina Tatton-Brown, Julien Thevenon, Magnus D Vigeland, Julie Vogt, Marjolaine Willems, Jonathan Zonana, D D D Study, Sarah F Smithson

**Affiliations:** 1 Department of Clinical Genetics, St Michaels Hospital, Bristol, UK; 2 School of Clinical Sciences, University of Bristol, Bristol, UK; 3 MRC Human Genetics Unit, MRC Institute of Genetics and Molecular Medicine, University of Edinburgh, Edinburgh, UK; 4 Institute of Human Genetics, University of Leipzig Hospitals and Clinics, Leipzig, Germany; 5 Institute of Medical Genetics, University Hospital of Wales, Cardiff, UK; 6 Service de Génétique Médicale, Hôpital de Hautepierre, Strasbourg, France; 7 Bristol Regional Genetics Laboratory, Southmead Hospital, Bristol, UK; 8 Northern Genetics Service, Newcastle upon Tyne Hospitals, Newcastle, UK; 9 Department of Clinical Genetics, Great Ormond Street Hospital, London, UK; 10 Department of Molecular and Medical Genetics, Oregon Health and Sciences University, Portland, OR, USA; 11 Centre de Génétique, CHU Dijon Bourgogne—Hôpital François Mitterrand, Dijon, France; 12 Department of Medical Genetics, Oslo University Hospital, Oslo, Norway; 13 Genetics and Metabolism, Pediatric Subspecialties, Carle Physicians Group, Urbana, IL, USA; 14 Northern Ireland Regional Genetics Service, Belfast City Hospital, Belfast, UK; 15 SW Thames Regional Genetics Service, St Georges University Hospital Trust, London, UK; 16 West Midlands Regional Genetics Service, Birmingham, UK; 17 Service de Génétique Médicale, Hôpital Arnaud de Villeneuve, CHRU de Montpellier, Montpellier, France; 18 Wellcome Trust Sanger Institute, Cambridge, UK

## Abstract

*PUF60* encodes a nucleic acid-binding protein, a component of multimeric complexes regulating RNA splicing and transcription. In 2013, patients with microdeletions of chromosome 8q24.3 including *PUF60* were found to have developmental delay, microcephaly, craniofacial, renal and cardiac defects. Very similar phenotypes have been described in six patients with variants in *PUF60*, suggesting that it underlies the syndrome. We report 12 additional patients with *PUF60* variants who were ascertained using exome sequencing: six through the Deciphering Developmental Disorders Study and six through similar projects. Detailed phenotypic analysis of all patients was undertaken. All 12 patients had *de novo* heterozygous *PUF60* variants on exome analysis, each confirmed by Sanger sequencing: four frameshift variants resulting in premature stop codons, three missense variants that clustered within the RNA recognition motif of PUF60 and five essential splice-site (ESS) variant. Analysis of cDNA from a fibroblast cell line derived from one of the patients with an ESS variants revealed aberrant splicing. The consistent feature was developmental delay and most patients had short stature. The phenotypic variability was striking; however, we observed similarities including spinal segmentation anomalies, congenital heart disease, ocular colobomata, hand anomalies and (in two patients) unilateral renal agenesis/horseshoe kidney. Characteristic facial features included micrognathia, a thin upper lip and long philtrum, narrow almond-shaped palpebral fissures, synophrys, flared eyebrows and facial hypertrichosis. Heterozygote loss-of-function variants in *PUF60* cause a phenotype comprising growth/developmental delay and craniofacial, cardiac, renal, ocular and spinal anomalies, adding to disorders of human development resulting from aberrant RNA processing/spliceosomal function.

## Introduction

In 2009, Verheij *et al.*^1^ reported overlapping interstitial microdeletions of chromosome 8q24 in two patients who had colobomata, congenital heart defects, limb abnormalities, developmental delay and seizures (MIM 615583). Dauber *et al.*^2^ later described five patients who also had microdeletions of 8q24.3 and similar phenotypes including ocular colobomata, microcephaly, developmental delay, short stature, craniofacial, cardiac and renal defects. All five patients had in common a 78-kb deleted region containing three genes: *SCRIB*, *NRBP2* and *PUF60*. A further patient with a missense variant in *PUF60*^2^ and a foetus with an 8q24.3 deletion encompassing *PUF60* occurring in association with an atrioventricular septal defect, a hypoplastic aortic arch, facial dysmorphism and other anomalies^3^ strongly implicated this single gene as the cause of the phenotype.

The poly-U-binding splicing factor gene (*PUF60)* encodes a nucleic acid-binding protein, which through interaction with other proteins such as SF3B4, regulates pre-RNA splicing and transcription.^2, 4^ In 2013 a single patient with an intragenic variant in *PUF60* was identified^2^ and recently five other cases have been reported.^5^ Here, we present clinical and molecular data from 12 additional patients with *de novo* variants in *PUF60* identified via exome sequencing undertaken for undiagnosed developmental disorders. The clinical features we observed in these patients and those previously published suggest that there is an emerging *PUF60-*related phenotype, although this is variable and may be difficult to recognize.

## Methods

### Patient ascertainment

Six of the affected patients were recruited via the Deciphering Developmental Disorders (DDD) study^6^ (http://www.ddduk.org) open to the UK NHS Regional Genetics Services. Five patients were recruited via locally based exome sequencing services by Clinical Geneticists in Norway, France and the United States and one via an exome sequencing study for patients with Cornelia de Lange Syndrome (CdLS; MIM 122470).^6^ All patients were assessed by their Clinical Geneticist who assisted with systematic detailed phenotyping. Patient growth centiles and *z*-scores were calculated using the UK WHO data (http://www.rcpch.ac.uk/growthcharts).

### Genomic analysis

Trio-based exome sequencing was undertaken for the six affected patients and their parents were identified via the DDD study.^7^ High-resolution analysis for copy number abnormalities using array-based comparative genomic hybridization was also performed. Putative *de novo* variants were identified from exome data using the DeNovoGear software^8^ and then validated by targeted Sanger sequencing. For patients 7–12, alternative but similar trio exome sequence approaches were used, for example, for patient 10 a proband-based exome sequencing as described previously,^8^ for patient 11 exome capture with Nextera Rapid Capture Exome Kit (Illumina Inc., San Diego, CA, USA) and sequencing with HiSeq4000 (Illumina). For patient 12, Agilent Clinical Research Exome Kit (Agilent Technologies, Santa Clara, CA, USA) identified exonic and periexonic fragments, followed by massive parallel next-generation sequencing. Analysis of exome data was undertaken with DeNovoGear^9^ or FILTUS,^10^ and for patient 11, raw data was processed using an end-to-end in-house database. Identified variants were annotated using standard databases and were filtered based on the established criteria. Detailed descriptions of the wet and dry laboratory pipelines are published elsewhere.^11^ For patient 12, analysis was performed with a custom-developed Xome Analyser (Gene Dx, Bethesda, MD, USA). Mean depth of coverage for the analysis was x121; and quality threshold (≥10x) was achieved for 95.5% of the target sequence. As for DDD patients, significant variants were confirmed by targeted Sanger sequencing. Variant data has been submitted to ClinVar (https://www.ncbi.nlm.nih.gov/clinvar/) – submission ID SUB2319251.

### Analysis of aberrant PUF60 splicing

Dermal fibroblasts were obtained from patient 8 (3781–3781) by skin punch biopsy and cultured in amnioMAX C-100 complete medium (Life Technologies, Carlsbad, CA, USA) as described previously.^12^ RNA was extracted from primary skin-derived fibroblast cell lines from patient 8 (3781–3781) and two sex-matched controls using RNeasy Kit (Qiagen, Hilden, Germany) and treated with *DNAse*I to eliminate genomic DNA, according to the manufacturer's instructions. Complementary DNA (cDNA) synthesis was carried out using random oligomer primers and AMV Reverse Transcriptase (Roche, Penzburg, Germany). The cDNA samples were resolved on an E-Gel electrophoresis system and extracted according to the manufacturer's instructions (Thermo Fisher Scientific, Waltham, MA, USA) to sequence the amplicons corresponding to the normal and mutant alleles. Sequences of primers used for cDNA amplification and Sanger sequencing of *PUF60* are available upon request.

## Results

The clinical features and *PUF60* variants in the 12 patients compared with the previously reported cases^2, 5^ are presented in Table 1.

### Variants

**Figure 1:**
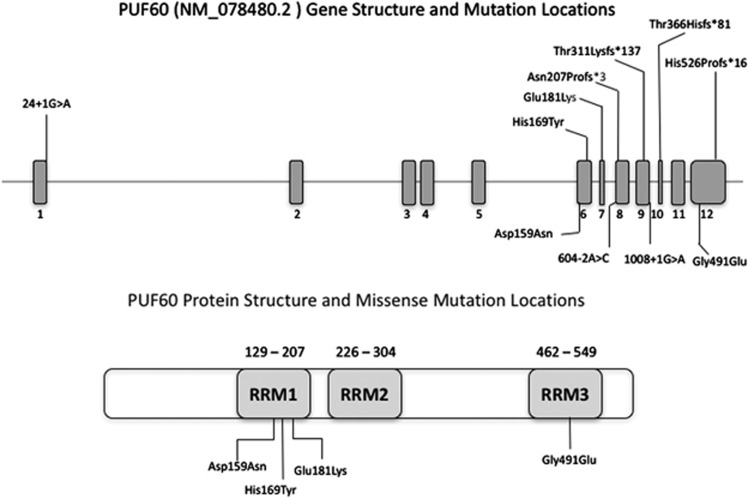
*PUF60* gene diagram (above) indicating mutations found in our cohort which cluster in the second half of the coding region, apart from the single variant in exon 1. Note recurrent splicing variant in exon 8. Below is pictured the known protein structure and location of RRM domains in relation to the missense variants.

**Figure 2:**
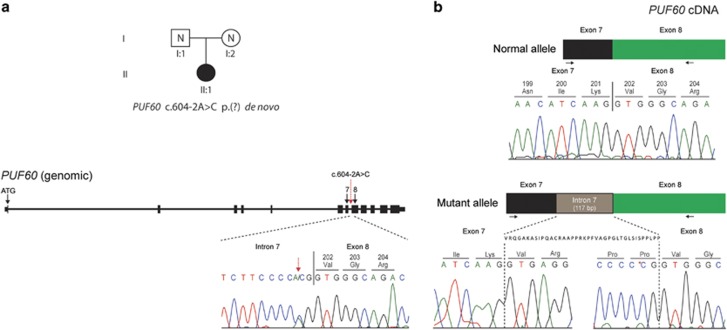
A *de novo* variant in *PUF60* results in aberrant splicing at exon 8 splice-acceptor site. (**a**) The heterozygous, *de novo PUF60* variant, c.604−2A>C was identified in patient II:1 through trio-based exome sequencing of family 3781. (**b**) Sequencing of skin-derived cDNA from patient II:1 (family 3781) showed normal splicing of exons 7 and 8 from one allele (top) and aberrant splicing with inclusion of the complete intron 7 from the mutant allele (bottom). Black arrows show the position of the oligonucleotide primers used for cDNA amplification and sequencing. The genomic context of the *PUF60* gene is shown, with exons indicated as black boxes. The location of the *PUF60* c.604−2A>C variant in intron 7 is indicated by a dotted red arrow, with the Sanger sequence trace from patient II:1 (family 3781) presented underneath. Variant nomenclature, exon numbering and the *PUF60* messenger RNA sequence are based on sequence accession numbers NM_078480.2 (mRNA) and NPs_510965.1 (protein) and GenBank accession number NG_033879.1.

The distribution of the heterozygous *de novo* variants (DNV) is shown in context of the genomic structure of *PUF60* in Figure 1. Nine of the DNV were predicted to cause loss-of function suggesting haploinsufficiency as the likely mutational mechanism. Four patients (1 (DDD275875),^13^ 5 (DDD270021), 9 and 11) had frameshift variants resulting in early stop codons. Five patients (2 (DDD273705), 7 (who had the most complex phenotype), 8 (3781–3781), 10 and 12) had essential splice-site variants. In patient 8 (3781–3781), a *de novo* c.604−2A>C variant occurred in the exon 8 splice-acceptor site and was predicted to alter splicing of *PUF60* mRNA. The effect of this variant was investigated as above: analysis of cDNA confirmed abnormal inclusion of the complete intron 7 in the mutant allele resulting in an apparent in-frame inclusion of 39 amino acids in the open reading frame of the transcript (Figure 2). It is thus not clear whether this essential splice-site variant will result in complete loss of function. Nonetheless, her phenotype is consistent with respect to developmental delay, short stature and cardiac involvement. Patient 12 was the only one to have a variant outside of the exon 6–12 region. The wild-type donor site is predicted to be disrupted resulting in skipping of exon 1; however, little is known about the exon 1 function (Human Splicing Finder score of −31.86 – probably affecting splicing; http://www.umd.be/HSF3/HSF.html).

Three patients in this study had missense variants located in regions encoding one of the three RNA recognition motif (RRM) domains of the protein (Alamut), c.541G>A(p.(Glu181Lys)) and c.475G>A(p.(Asp159Asn)) are located in RRM1 and c.1472G>A(p.(Gly491Glu) in RRM3. CADD scores^14^ are shown in Table 1 and support effect on function of all three variants.

None of the *PUF60* variants we identified are listed in the Exome Variant Server, Exome Aggregation Consortium or the dbSNP population databases.

### Growth

Birthweight was normal except for patients 6 (DDD263362; *Z*-score −2.14), 8 (3781–3781; *Z*-score −2.11) and 11 (*Z*-score −3.24). Body weight in childhood remained in the normal range in all patients apart from patients 7 and 8 (3781–3781; *Z*-scores −4.47 and −3.52 respectively) in both of whom other growth parameters were also significantly reduced. Nine patients had short stature defined as height below 5th centile and five had *Z*-scores below −2. Head circumference was proportionate to stature, apart from in five patients (1 (DDD275875), 6 (DDD263362), 7, 10 and 11) who had true microcephaly (*Z*-scores −2.48, −4.22, −2.09, −2.99, −2.53 respectively).

### Musculoskeletal

**Figure 3:**
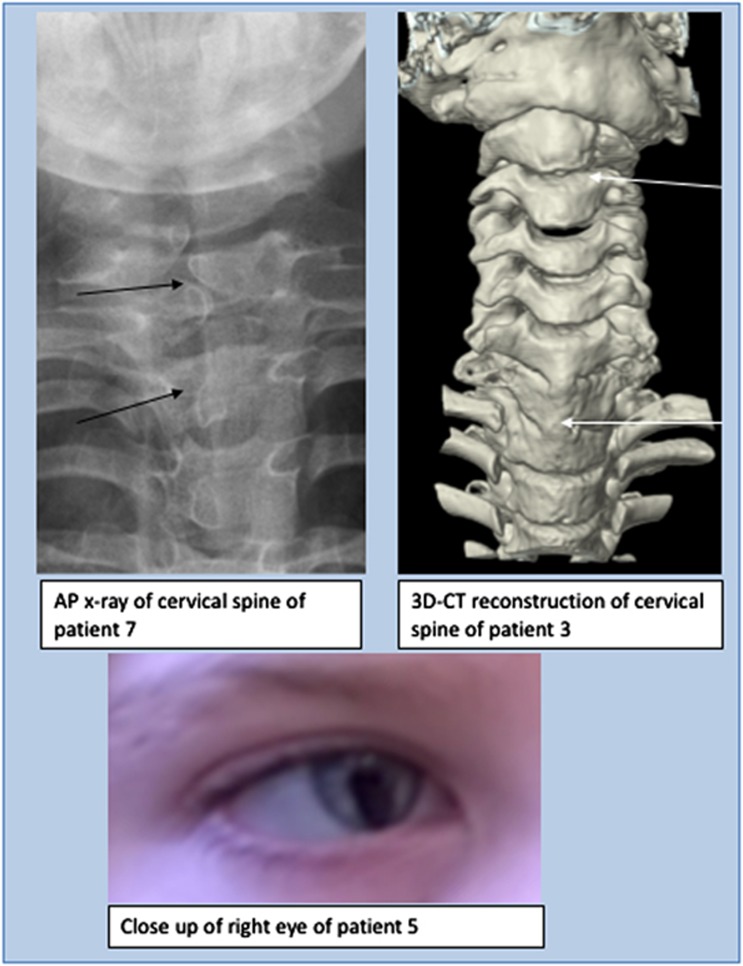
Spinal features (above): AP x-ray of cervical spine of patient 7 demonstrating hemivertebrae (left) and 3D-CT reconstruction of cervical spine of patient 3 demonstrating abnormalities of articulation of atlas with C1, vertebral bodies of C2/C3 and fusion of bodies of C6/C7/T1 and ocular features (below): right iris coloboma in patient 5.

Skeletal abnormalities, especially in the spine, were seen in seven patients. In patient 3 (DDD271317), there was fusion of the whole vertebral bodies of C6 and C7 and the anterior part of C5 (Figure 3) and the posterior spinous processes of T6, T7 and T1 were unusually prominent. His cervical spine stability requires neurosurgical monitoring. Patient 7 had several cervical and thoracic hemivertebrae, spina bifida, thoracic kyphosis, bilateral rudimentary ribs at C7 and pectus excavatum (possibly exacerbated by sternotomy during cardiac surgery). In patient 9, the conus terminated at the L2-L3 disc space and the filum, 2 mm in width, extended from L3 to S2 associated with posterior osseous dysraphism of the sacrum. Patients 6 (DDD263362), 10 and 12 had pectus excavatum. Patient 1 (DDD275875) had a lower thoracic scoliosis and pes planus. He also had shoulder subluxation and generalized joint laxity, which was a common feature in the cohort. Patients 2 (DDD273705), 7, 9, 10 and 12 also had joint hypermobility; in one case this was associated with bilateral hip and interphalangeal joint dislocations. Digital anomalies in the group included unilateral preaxial polydactyly of the left hand with a broad proximal phalanx and duplicated distal phalanx (patient 7), short broad hands with bilateral fifth finger clinodactyly (patient 5 (DDD270021), left talipes, broad thumbs/halluces, fifth finger clinodactyly, 2–3 toe syndactyly with an overriding second toe on the left (patient 6 (DDD263362)) and bilateral hypoplasia of the fifth fingers (patient 9).

### Auditory

Two patients had very narrow and easily occluded external auditory meatus, two had severe otitis media with episodic conductive hearing loss and two wear hearing aids for bilateral conductive hearing loss.

### Development

All patients had mild to moderate global developmental delay. Most walked by 24 months and the latest did so by 30 months (patient 12 was not yet walking at 24 months). Most spoke their first word by 30 months. Three patients had difficulties forming a pincer grip and with fine motor coordination for activities such as handwriting. For those who are currently of school age, all receive additional educational support and four attend either a special needs school or class. Some episodes of difficult or immature behaviour including attention deficit disorder, head-banging, self-injury or temper tantrums were reported by parents of five patients.

### Neurology

Patient 6 (DDD263362) had a stormy postnatal course during which bilateral intraventricular haemorrhages occurred, later associated with mild diplegia. An MRI brain scan showed periventricular leukomalacia, a thin corpus callosum and a cyst at the right cerebellopontine angle. Patient 7 had a cardiac catheterization at 9 months of age and following a right middle cerebral artery occlusion had a left-sided hemiparesis and right facial weakness. A brain MRI scan of patient 11 showed mild periventricular gliosis. Patient 12 had an MRI scan, which showed cerebral ventriculomegaly, partial agenesis corpus callosum and loss of white matter in the periventricular regions. Two patients have developed seizures.

### Facial features

**Figure 4:**
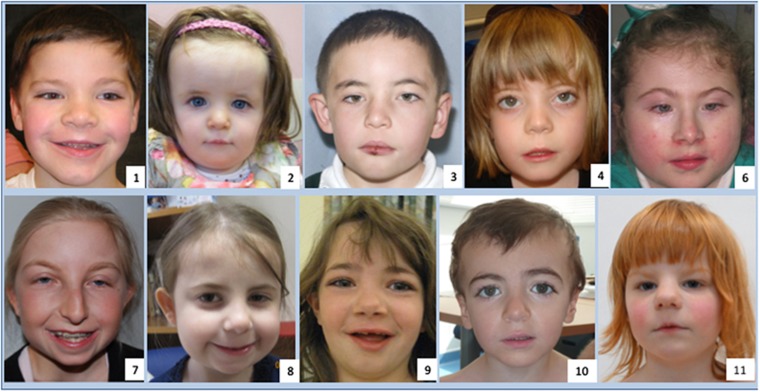
Faces of patients (1, 2, 3, 4,6, 7, 8, 9, 10 and 11) with *PUF60* variants, illustrating the thin upper lip, long philtrum, micrognathia and the flaring of eyebrows and narrow almond-shaped palpebral fissures, which are variably present. Patients 6 and 8 were assessed for CdLS and patients 7 and 10 had *CREBBP* analysis because of facial similarities with Rubinstein–Taybi syndrome. Patient 9 was investigated for Coffin–Siris syndrome.

The facial features (Figure 4) included a short neck in five >patients, a high forehead in six, micrognathia in four, a long philtrum in five, bushy eyebrows in four and a thin upper lip in four patients. One patient had synophrys and long eyelashes, another had bilateral preauricular pits and a third, a right accessory auricle and skin tag on the contralateral side of the neck. Two patients had facial features initially suggesting CdLS. Two other patients were tested for mutations in *CREBBP* and *EP300* initially. One patient was tested for Coffin–Siris syndrome.

### Other

Recurrent ocular, cardiac and renal features occurred and are detailed in Table 1. Five patients had hypertrichosis of the face. Two patients had macrodontia, one with ectopic teeth and one early secondary dentition. Early feeding problems were very common: in one patient a gastrostomy was required from 9 months to 7 years of age and three patients were prone to aspiration episodes and complications in infancy, this resolved in two of them by late infancy.

## Discussion

The clinical and molecular data we observed in 12 patients suggest that variants in *PUF60* cause a syndrome characterized by short stature, developmental delay, dysmorphic facial features and structural malformations of the heart, eye and variably other organs. We are aware of 14 *PUF60*-related published cases: six with intragenic *de novo* missense variants^2, 5^ and eight with chromosome 8q24.3 deletions encompassing *PUF60*.^1, 2, 3^ The pattern of their congenital malformations and perturbation of growth and development shows some common themes.

Comparison of our patients with the six previously published for whom data are available (Table 1) suggests that short stature, relative microcephaly and developmental/cognitive delay are consistent findings. The only exception is patient 12 who had macrocephaly, hydrocephalus and normal stature, and in whom the variant was at the exon 1 boundary. A previously published patient had an identical variant^5^ but their phenotype was more typical, thus predictions on phenotype based on position of specific variants are not possible at this stage. We observed other similarities: a high proportion of cases overall had abnormal segmentation of the vertebrae, which occurred at different levels of the spine. One of our patients and two published patients^5^ had cervical vertebral fusion/abnormal cervical spines. Other authors found more distal lesions such as fusion of L5-S1, sacral dysplasia and agenesis of the coccyx,^2^ and other patients, including patients 7, had hemivertebrae in the thoracic and lumbar spine. We and others found minor developmental abnormalities of the distal limbs, including brachydactyly of the fifth fingers, clinodactyly and preaxial polydactyly. Ocular coloboma involving the anterior segment of the eye, choroid and/or the retina, were described in three previous as well as in two of our patients. Renal malformations such as ectopic fused kidneys, pelvic kidney, a unilateral polycystic kidney and renal hypoplasia or unilateral agenesis have been observed,^2, 5^ and in our cohort there was one case each of a unilateral kidney and a horseshoe kidney. Five of our patients had significant congenital heart defects and other authors^5^ have also reported ventricular septal defects in combination with truncus arteriosus coarctation or the aorta and bicuspid aortic valve^2^ and atrioventricular septal defects and hypoplastic aortic arch.^3^

While all 26 patients with deletions or variants of *PUF60* had developmental delay and intellectual disability, those with deletions appear to be more severely affected.^1, 2^ The reported microdeletions of chromosome 8q24.3 may include other currently unknown genes that contribute to neurological and cognitive development. It is interesting that seizures were observed in our patients as well as in previous reports. Feeding difficulties and recurrent respiratory infections were common to some patients in both groups.

Previous authors commented on phenotypic similarity of *PUF60*-deleted patients with *SF3BF4*-related Nager syndrome (MIM 154400) and *EFTUD2*-related mandibulofacial dysostosis (MIM 610536) and suggested that PUF60 deficiency can be considered within the spectrum of craniofacial disorders resulting from spliceosome malfunction.^15^ However, coloboma, optic nerve hypoplasia and facial hirsutism have not hitherto been reported in syndromes ascribed to spliceosomal dysfunction. We and others noticed that some patients had features reminiscent of CdLS, particularly where facial hypertrichosis and prominent eyebrows were present. Comparison of the facial features in all patients with *PUF60* deletions or variants where data were available indicated that micrognathia, a long philtrum and thin upper lip, synophrys, preauricular pits and cranial asymmetry were common findings. Additionally, two of our patients had very narrow external auditory canals, consistent with abnormal branchial arch development during embryogenesis, also thought to be a process disturbed in mandibulofacial dysostosis and Nager syndromes; however, the facial dysmorphology of patients with *PUF60* variants is far more subtle than in *EFTUD2* and *SF3B4*-related phenotypes and mandibulofacial dysostosis was not present in the patients we report. Furthermore, it is interesting to note that none of the patients were clinically suspected of a known spliceosomopathy. We conclude that the facial features associated with *PUF60* might be recognized independently, but the diagnosis is more likely to be considered in a child with short stature, developmental delay and additional malformations described.

There is accumulating evidence from this and other phenotypic studies that *PUF60* is important in human embryonic development. PUF60 belongs to the RRM half pint family and contains three RRM domains. Consistent with a developmental role, *PUF60* encodes a DNA- and RNA-binding protein that is involved in diverse nuclear processes such as pre-mRNA splicing and regulation of transcription. During pre-mRNA splicing, PUF60 interacts with U2AF2 to promote splicing of an intron with a weak 3' splice site^4^ and has a role in alternative splicing.^2^ PUF60 is one of the non-core components that can ally with the U2 spliceosomal small nuclear ribonucleoprotein particles, or U2 spliceosome complex.^16^ Finally, PUF60 has been associated with the transcriptional repression of MYC through association with FUBP1. The mechanism by which reduced *PUF60* expression affects cellular functions are not yet understood and beyond the scope of this study. It is an interesting though that the *PUF60*-related phenotype is more diverse than some others determined by spliceosomal genes, where very specific and recognizable malformations arise.^17^ Presumably, specific tissues are PUF60 dose-sensitive at specific times during early human development.

The similar phenotypes in 8q24.3 deletions encompassing the entire *PUF60* gene and the point variants in our series, including both missense and null variants, suggest that haploinsufficiency is the common mechanism in all. Loss-of-function variants are predicted to result in altered dosage of different PUF60 isoforms and consequently abnormal splicing of target genes.^2^ Variants in this gene would therefore be expected to have widespread phenotypic effects. Previous researchers found that patient-derived cells demonstrated expression of a truncated specific isoform of PUF60.^2^ They also showed that suppression of puf60 in developing zebrafish resulted in reduced body length, microcephaly, craniofacial defects and cardiac anomalies. Additionally, they found evidence that suppression of the *scrib* gene (syntenic and adjacent to *PUF60* in humans) caused defects such as colobomata and renal anomalies, and thus concluded that some features of the 8q24.3 deletion phenotype might be due to this contiguous gene. Our data together with the other six reported cases suggest that PUF60 deficiency itself could account for these features in humans. We anticipate that more patients with *PUF60* variants will be described, which, in association with functional studies, will help to delineate the characteristics of this complex syndrome.

## Conclusions

Chromosome 8q24.3 deletions have been associated with a phenotype encompassing microcephaly and short stature, developmental delay, colobomata, craniofacial, skeletal, cardiac and renal anomalies. Our findings demonstrate a very similar pattern of mild or moderate intellectual disability and physical characteristics observed in patients with variants in *PUF60*. We suggest that loss or reduction of expression of *PUF60* results in a complex human phenotype with subtle facial features and a consistent pattern of congenital malformations, especially involving the heart and spine.

## Figures and Tables

**Table 1:**
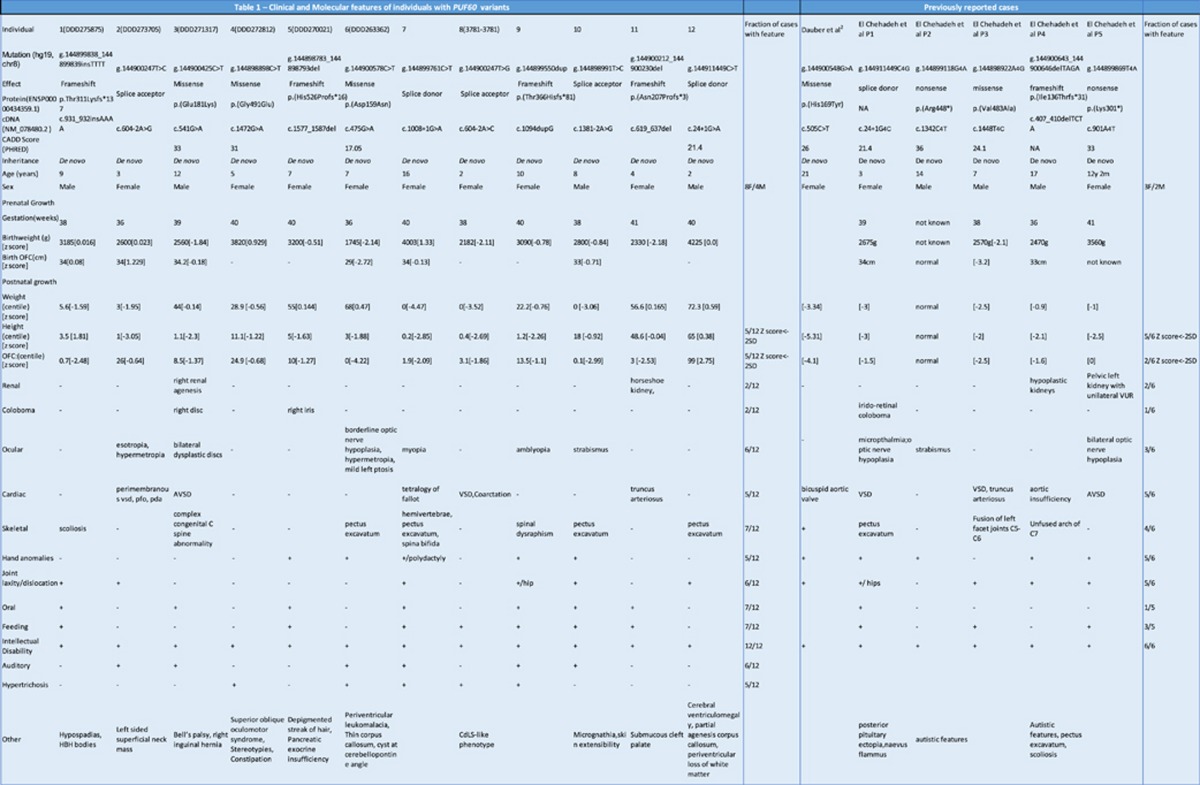
Clinical and genetic findings in patients with *PUF60* variants

Abbreviations: AVSD, atrioventricular septal defect; CADD, Combined Annotation Dependent Depletion; CdLS, Cornelia de Lange Syndrome; HBH, haemoglobin H; OFC, occipitofrontal circumference; PDA, patent ductus arteriosus; PFO, patent foramen ovale; VSD, ventricular septal defect.

Table lists presence of phenotypic features in each patient in our cohort, as well as in previously reported patients along with total frequency of each phenotypic feature. Mutation data including genomic location, predicted effect on protein are listed for all patients with CADD score^14^ calculations for all missense mutations and where previously reported. (CADD is a framework that integrates multiple existing annotation tools to give one scaled score of deleteriousness for a variant. A CADD score of 20 means that a variant is among the top 1% of deleterious variants in the human genome. A score of 30 means that the variant is in the top 0.1% and so forth.).
